# The Medical Association of Central New York

**Published:** 1894-11

**Authors:** Edward B. Angell

**Affiliations:** Secretary, of Rochester, N. Y.


### THE MEDICAL ASSOCIATION OF CENTRAL NEW YORK.

Reported by EDWARD B. ANGELL, M. D., Secretary, of Rochester, N. Y.

The twenty-seventh annual meeting of the Medical Association of Central New York was held at the Academy of Medicine Parlors, in the City of Buffalo, N. Y., Tuesday, October 16, 1894. The association was called to order at 10 o'clock by the president, Dr. H. L. Elsner, of Syracuse. The minutes of the last meeting were read and approved.

The president then announced the following committees:

On Business—Drs. Lucien Howe, of Erie; Geo. E. Clark, of Onondaga; A. S. Hall, of Cayuga.

On Credentials—Drs. John O. Roe, of Monroe; A. L. Benedict, of Erie.

On Reception—Drs. Floyd S. Crego, M. D. Mann, Roswell Park, Chas. G. Stockton, A. A. Hubbell, of Erie County.

Dr. F. S. Crego, of Buffalo, vice-president, then took the chair while the president's address, on Clinical and Bacteriological Aspect of Mixed or Concurrent Infection in Pulmonary Tuberculosis, was delivered by Henry L. Elsner, M. D., of Syracuse, N. Y.

Dr. Roe, of Monroe, moved that a vote of thanks be tendered to the president for his very able and instructive address. Carried.

Dr. Howe, as chairman of the business committee, then announced the order of business, and stated that on account of the length of the program it would be necessary to limit the reader of each paper to ten minutes, and its discussion to two and a half minutes.

Papers were then read as follows:

1. Hydrotherapy of Appendicitis, by Dr. George E. Clark, of Skaneateles, N. Y.; discussed by Dr. Jacobson, of Syracuse, N. Y. 2. Some Points in the Ethiology, Pathology and Indicated Treatment of Diphtheria, by A. A. Young, M. D., of Newark, N. Y.; discussed by Drs. Roe, Jacobson and Thornbury and, in closing, by Dr. Young. 3. The Home Treatment of Hay Fever, by A. L. Hall, M. D., of Fair Haven; discussed by Dr. Roe and, in closing, by Dr. Hall. 4. A New Microörganism Discovered in Pork, by F. J. Thornbury, M. D., of Buffalo, N. Y. 5. Atonic Dyspepsia, by A. L. Benedict, M. D., of Buffalo, N. Y.; discussed by Dr. Jones and, in closing, by Dr. Benedict. 6. The Treatment of Lactic Acid Excess in the Stomach, by A. A. Jones, M. D., of Buffalo, N. Y.; discussed by Drs. Benedict and Zimmer and, in closing, by Dr. Jones. 7. Exhibition of a Neurological Bust, devised by Wm. C. Krauss, M. D., of Buffalo, N. Y. 8. A Case of Nephrectomy, by Herman Mynter, M. D., of Buffalo, N. Y.

Recess was then taken, and the members proceeded to the Genesee Hotel, where they were entertained at dinner as the guests of the Medical Society of the County of Erie.

AFTERNOON SESSION, 3 P. M.

The report of the treasurer was read and showed a balance on hand of $214.05. Approved.

The election of officers for the ensuing year resulted as follows: President, Dr. Floyd S. Crego, of Buffalo; first vice-president, Dr. William S. Cheeseman, of Auburn; second vice-president, Dr. E. L. Mooney, of Syracuse. The secretary and treasurer hold their offices for another year. Drs. E. B. Potter, E. B. Angell and A. A. Young were elected delegates to the American Medical Association, and the secretary was authorized to give the necessary credentials to any member desirous of attending the meeting.

The report of the publication committee was presented by the secretary, announcing the arrangement made with the Buffalo Medical and Surgical Journal for the publication of the proceedings. The committee had considered it unadvisable this year to print a revised constitution and list of members. The report was accepted and the committee discharged.

The president then appointed as publication committee for the ensuing year the secretary, Dr. Angell, and the treasurer, Dr. Mercer, to which committee Dr. Wm. C. Krauss, of Erie County, was added. The committee was authorized to publish at their discretion the revision of the constitution with list of members.

The chairman of the business committee then read two letters of regret, one from Dr. M. D. Mann, of Buffalo, and the other from Dr. L. A. Weigel, of Rochester.

Papers were then read as follows:

9. Report of two Cases of Extra-uterine Pregnancy—Operation—Recovery, by Herman E. Hayd, M. D., of Buffalo, N. Y. 10. The Pathology of Trichinosis—Original Observations—Illustrated, by F. J. Thornbury, M. D., of Buffalo, N. Y. 11. Observations Upon an Anencephalic Monster, by E. B. Angell, M. D., and S. L. Elsner, M. D., of Rochester, N. Y. 12. Discussion Upon the Management of Occipito-posterior Positions—participated in by Drs. Van Peyma and Frederick, of Buffalo, N. Y. 13. Report of a Recent Case of Abdominal Pregnancy—Laparatomy—Recovery, by H. T. Williams, M. D., of Rochester, N. Y.; discussed by Dr. Mynter. 14. Atrophic Changes in the Optic Nerve, the Result of Nervous Disease, by A. A. Hubbell, M. D., of Buffalo, N. Y.; discussed by Drs. Howe, Crego and, in closing, by Dr. Hubbell. 15. Dangers of the Intra-uterine Tampon, by W. E. Ford, M. D., of Utica, N. Y.; discussed by Dr. Williams. 16. Septic Inflammation of the Cerebral Sinuses, by Roswell Park, M. D., of Buffalo, N. Y. 17. Uterine Hydatids, by H. D. Ingraham, M. D., of Buffalo, N. Y.; discussed by Dr. Van Peyma. 18. A Report of One Year's Work in Abdominal and Gynecological Surgery at the Buffalo Women's Hospital, by C. C. Frederick, M. D., of Buffalo, N. Y. 19. Faradic High Tension Coils and Currents, by Dr. F. W. Ross, of Elmira, N. Y.; discussed by Drs. Park, Ford, Crego and, in closing, by Dr. Ross. 20. Report of a Case of Sarcoma of the Abdomen Cured by the Toxines of Erysipelas, by Dr. H. Mynter of Buffalo, N. Y.; discussed by Dr. Ingraham, and Dr. Mynter in closing.

The following papers were then read by title and ordered printed in the transactions:

21. The Use and Abuse of Mechanical Appliances, by L. A. Weigel, M. D., Rochester, N. Y. 22. A Case of Bruised Nerve, by John Van Duyn, M. D., Syracuse. 23. The Treatment of Sleeplessness Without the Use of Narcotics, by F. S. Crego, M. D., of Buffalo. 24. Enteroclysis in the Various Forms of Enterocolitis and the Zymotics Generally, by F. W. Bartlett, M. D., of Buffalo. 25. Intra-abdominal Shortening of the Round Ligaments for Retro-displacements of the Uterus, by M. D. Mann, M. D., of Buffalo. 26. A Few Cases of Puerperal Convulsions, by F. W. Sears, M. D., of Syracuse.

Under the head of miscellaneous business, Dr. W. C. Phelps, of Buffalo, made the following remarks:

Mr. President—I have a letter which I received from Mr. Tracy C. Becker, who is a lawyer in this city and is quite interested in medico-legal affairs, being one of the authors of a book that is being published by William Wood & Co.; he was also a delegate to the late Constitutional Convention. He is interested in an amendment adopted by the convention abolishing the office of coroner. He tells me the scheme that he and others have on hand is to have a law passed by the legislature which provides for the same duties that have formerly been performed by the coroner, but limiting the eligibility to hold that office to members of the medical profession, so that no one but physicians can hold the office. As a preliminary to that, the office of coroner must be abolished, and that can only be done by altering the constitution of the state. He thinks, as a lawyer, and as physicians I think we know the same thing, that the office of coroner has been a good deal of a farce and it is very reasonable that when a person dies, any official investigation should be conducted by a doctor, and that is what the medico-legal society and the law society and others are working for. This amendment is bunched up to be voted on separately, not along with the other amendments, especially the apportionment, with the amendment which prohibits the selling of pools on race tracks. The race track people are raising thousands of dollars. I saw today that it was reported that a gentleman of Buffalo had sent $50,000 as a fund to prevent the adoption of this branch of the constitutional amendments. Mr. Becker suggests that physicians all over the state, and he wished me to bring it up before this association especially, should be urged to vote for this amendment, and also get all their patients that they can to vote for it, with the idea of finally throwing this office of coroner into the hands of the medical profession. He suggests that the secretary of this association send a note to each member urging him to vote for this special amendment. I would, therefore, make that motion,

"That the secretary be requested to send a note to each member requesting him to vote for this amendment to the Constitution of the State of New York."

Dr. CREGO: I would add as an amendment, "that this Society endorses the action of the Constitutional Convention in passing such a resolution." I think that would give a little more weight to it. I think we are all in favor of it.

Dr. PHELPS: I accept the amendment.

The resolution, as amended, was seconded and carried, and the secretary directed to communicate the action of the association to each member.

Dr. MOONEY moved that a vote of thanks be extended to the Medical Society of the County of Erie for the very pleasant manner in which the Association has been entertained.

Seconded and carried.

The secretary announced the names of the following physicians who had complied with the requirements of the by-laws and moved their election to permanent membership: Drs. John J. Twohey, A. L. Benedict, F. W. Bartlett, Wm. C. Krauss and A. J. Martin, of Erie County; John W. Flick, Frederick Remington, W. J. Howe, Thomas Jameson, Sumner Hayward, E. B. Potter, John D. Briggs, J. H. Schopp, Charles R. Barber, and S. W. Rose, of Monroe County; John H. Batt and F. L. Stebbins, of Ontario County; R. W. Bamler, of Orleans County; M. E. Carver and M. Alice Brownell, of Wayne County.

The motion was seconded and carried and they were declared elected permanent members.

President ELSNER then addressed the association as follows:

*Members of the Central New York Medical Association:*

In declaring this meeting adjourned I wish personally to express my deep sense of obligation to the members of the Medical Society of the County of Erie, to all gentlemen who have taken part here today, to our secretary for his untiring efforts in making this, our twenty-seventh annual meeting, a success. I feel that this has been in every sense of the word a successful meeting. I do not know where we could go and find as many enthusiastic workers as have worked today with us to make this meeting a complete success, and I hope that next year when we meet in Syracuse, we will have as large, if not a larger gathering, and that all of you who have been here today will be with us there, the third Tuesday in October, 1895.

The association then adjourned.

